# Flexible Solar Interfacial Evaporators with Photocatalytic Function for Purification of High-Salinity Organic Wastewater

**DOI:** 10.3390/nano15080632

**Published:** 2025-04-21

**Authors:** Yucheng Li, Xia Zhao, Tao Hu, Lingxiao Li, Xiaopeng Huang, Junping Zhang

**Affiliations:** 1 Department of Environmental Engineering, College of Petrochemical Engineering, Lanzhou University of Technology, Lanzhou 730050, China; lao17629086530@163.com; 2 Research Center of Resource Chemistry and Energy Materials, Lanzhou Institute of Chemical Physics, Chinese Academy of Sciences, Lanzhou 730000, China; hutao@licp.cas.cn; 3 State Key Laboratory of Featured Metal Materials and Life-Cycle Safety for Composite Structures, School of Resources, Environment and Materials, Guangxi University, Nanning 530004, China; lilx@gxu.edu.cn; 4 Department of Chemical Engineering, College of Petrochemical Engineering, Lanzhou University of Technology, Lanzhou 730050, China; 18419210628@139.com

**Keywords:** solar-driven interfacial evaporation, photothermal, photocatalysis, metal organic framework, desalination

## Abstract

Solar-driven interfacial water evaporation technology coupled with photocatalytic function is regarded as an emerging approach for treating high-salinity organic wastewater, but it remains challenging to design high-performance solar evaporators with excellent photocatalytic properties. Here, we designed a two-dimensional flexible solar interfacial evaporator with photocatalytic function for the purification of high-salinity organic wastewater. The solar evaporator was prepared by the deposition of Cu-based metal organic frameworks (Cu-MOFs) onto a polyester fabric by solvothermal reaction, during which graphitic carbon nitride was also deposited as carried by Cu-MOFs. The solar evaporator achieves an outstanding evaporation rate of 1.95 kg m^−2^ h^−1^ for simulated seawater (3.5 wt% NaCl) under 1 sun. The evaporator also shows efficient evaporation performance and salt resistance for high-concentration saline water due to its outstanding water transport capacity and efficient light absorption ability. Furthermore, salt ions and organic pollutants can be simultaneously removed from high-salinity organic wastewater by the evaporator due to the synergistic effects of adsorption, the photothermal effect and photocatalytic performance. This study successfully integrated photocatalytic technology with solar-driven interfacial evaporation, extending the multifunctionality of solar evaporators for treating high-salinity organic wastewater.

## 1. Introduction

Freshwater resources constitute merely 2.5% of the global water supply, of which only ~1% is readily accessible for human consumption and industrial use. However, the steadily increasing global population and industrial growth are generating large volumes of simulated high-salinity wastewater, intensifying concerns over water scarcity [1,2,3,4,5]. Therefore, to address the freshwater crisis, there is an urgent requirement to develop environmentally sustainable and low-carbon technologies. Solar-driven interfacial evaporation, an emerging photothermal desalination technology that localizes vapor generation at the liquid–air interface, has demonstrated remarkable potential for freshwater production. This technology is particularly promising due to its superior evaporation efficiency, excellent photothermal conversion capability and inherent environmental friendliness, making it one of the most viable solutions to global water scarcity challenges [6,7,8]. Furthermore, solar-driven interfacial evaporation technology enhances the evaporation rate by modifying the evaporator structure to increase the photothermal contact area and solar light utilization efficiency. However, some of the evaporators reported to date suffer from issues such as low evaporation efficiency and high thermal losses [9,10,11,12]. In particular, most current evaporators are ineffective in removing organic pollutants from high-salinity wastewater. During the photothermal evaporation process, water-soluble small-molecule organic contaminants may be transferred into the condensate along with the vapor. Creative ideas have been developed for solving this scientific challenge by synergistically coupling photocatalysis with photothermal reaction to produce a photocatalytically coupled solar-driven interface evaporator. 

Photocatalytic technology is widely used in environmental treatment, especially for treating organic pollutants, due to its mild reaction conditions, high degradation rate, wide applicability and energy saving [13,14]. The photogenerated electrons and holes excited by ultraviolet and visible light in photocatalysts can facilitate the redox reactions of water molecules, thereby facilitating the degradation of organic pollutants [14,15,16,17]. However, photocatalysts such as graphitic carbon nitride (g-C_3_N_4_) are typically in powder form, making recovery after use exceedingly difficult [18,19,20]. The integration of photocatalysts with solar-driven evaporators can effectively address this issue. Solar evaporators can absorb solar energy in the visible light region, accelerate water evaporation to produce clean water and meanwhile promote photocatalytic reactions [16,21]. The combination of both technologies can provide significant assistance in the treatment of high-salinity wastewater. Li et al. [19]. constructed a novel composite solar interface evaporation device (3D-2D-3D BiOI/porous g-C_3_N_4_/graphene hydrogel) by a two-step synthesis method. The evaporator demonstrated the integration of photocatalysis and photothermal action. The degradation rates for the organic pollutant methyl blue (MB) and the antibiotic levofloxacin chloride (LVF) were 8.12 and 3.73 times higher than those of bismuth xyiodide (BiOI) in the stationary state and 1.54 and 1.678 times higher than those of BiOI in the flowing state, respectively. Furthermore, the degradation rates remained stable over a period of 30 h. Lv et al. [21]. prepared a multiple-function CuCoNi evaporator. The evaporator had excellent evaporation performance (1.91 kg m^−2^ h^−1^ under 1 kW m^−2^) and could degrade both non-volatile organic pollutants and volatile organic pollutants, and the degradation rates of both were greater than 98.2%.

Here, spherical g-C_3_N_4_ and Cu-based metal organic frameworks (Cu-MOFs) were deposited on a polyester (PET) fabric by a solvothermal method to fabricate the solar evaporator coupled with photocatalytic functionality. Polyester (PET) fabrics, due to their availability, mechanical strength and hydrophilicity, have been widely used in solar-driven interfacial evaporation. MOFs, owing to their high specific surface area and superior light absorption properties, are regarded as ideal candidates for photothermal materials [22]. The PET@Cu-MOF/g-C_3_N_4_ evaporator, designated as PCG evaporator, exhibits excellent photocatalytic activity. Under light irradiation conditions, g-C_3_N_4_ generates superoxide anion radicals (O_2_^−^) and hydroxyl radicals (-OH) to degrade the organic pollutant MB with a degradation rate of 95.4% [18,20,23]. Moreover, the PCG evaporator demonstrates excellent evaporation performance, with an evaporation rate of 1.95 kg·m^−2^·h^−1^ in a 3.5 wt% NaCl solution under 1 sun intensity, an evaporation efficiency (η) of 94.4% and stable evaporation performance. The Na^+^, K^+^, Ca^2+^ and Mg^2+^ concentrations in the condensation water are below the limits defined in the water quality standards set by the WHO.

## 2. Materials and Methods

### 2.1. Materials

Dicyandiamide (DCD), 2,4,6-trichloro-1,3,5-triazine ((ClCN)_3_), Cu(NO_3_)_2_ and 2,3,6,7,10,11-hexahydroxytriphenylene hydrate (HHTP) were purchased from Shanghai Aladdin Biochemical Technology Co., Ltd., Shanghai, China. MB, N, N-dimethylformamide (DMF) and acetonitrile were purchased from China National Medicines Co., Ltd., Beijing, China. Analytical-grade chemicals and ultrapure water were used throughout the experiments.

### 2.2. Preparation of g-C_3_N_4_

A solvothermal method recommended in the literature was used to prepare g-C_3_N_4_ [18,19,20]. A total of 2.76 g of (ClCN)_3_ and 0.62 g of DCD were added to 60 mL of acetonitrile solution, ultrasonicated in a sealed system for 10 min and stirred at 500 rpm for 24 h at room temperature (25 °C). After stirring, the mixture was slowly transferred into a 100 mL autoclave, which was then subjected to a solvothermal reaction at 180 °C for 48 h. The resulting product was filtered and washed sequentially with acetonitrile solution, distilled water and absolute ethanol. Acetonitrile washing was performed 1–2 times, and distilled water washing continued until the supernatant pH reached neutrality (approximately 9–14 times), followed by two washes with absolute ethanol. The as-prepared g-C_3_N_4_ was vacuum-dried at 60 °C for 12 h, yielding a reddish-brown powder (Figure S1).

### 2.3. Fabrication of PCG Evaporators

Next, 0.0582 g of Cu (NO_3_)_2_, 0.039 g of HHTP and 0.1 g of g-C_3_N_4_ were dispersed into 6 mL of an DMF/water solution (1/1, *v*/*v*) via stirring for 30 min. Subsequently, the uniform Cu-MOF/g-C_3_N_4_ mixture was impregnated and adsorbed onto PET. Lastly, a solvothermal reaction was carried out at 85 °C for 15 h to produce the PCG evaporator.

### 2.4. Interfacial Solar Evaporation Performance Tests

The experiment was conducted in a simulated sunlight environment (1 kW m^−2^) to assess the photothermal evaporation performance of diverse samples. All evaporation performance tests were conducted under controlled laboratory conditions using a real-time monitoring system integrating mass measurement and infrared thermal imaging. The mass measurement system was made up of a solar simulator, an analytical balance and a computer for recording time-dependent quality changes in water due to photothermal evaporation. The infrared thermal imaging system was composed of an infrared camera. The intensity of solar irradiation was measured by a full-spectrum optical power meter. The test environment conditions were maintained at 25 ± 3 °C, and the relative humidity (RH) was 30 ± 3%. The evaporation efficiency (η) was calculated according to the following Equation (1).

η = m_evap_h_lv_/q_i_C_opt_ (1)

Here, m_evap_ refers to the vapor mass flux by subtracting the water evaporation rate observed in dark conditions, C_opt_ denotes the optical concentration and q_i_ represents the standardized direct solar irradiance (1 kW m^−2^). The parameter h_lv_ quantifies the total enthalpy variation during the liquid–vapor phase transition of water, derived from Equation (2).

H = λ + C∆T(2)

where *λ* denotes the phase-change latent heat, *C* designates the specific heat capacity of water (4.2 kJ kg^−1^ K^−1^) and Δ*T* quantifies the temperature rise at the liquid–solid interface.

### 2.5. MB Removal Experiment Tests

A laboratory-designed device was used to conduct simulated MB removal tests. The concentration of MB was monitored using a UV-Vis spectrophotometer (T2602, Youke Instrument Co., Ltd., Shanghai, China). The experiment was conducted under simulated sunlight intensity conditions of 1 sun (1 sun = 1 kW m^−2^). As shown in Figure S8, stirring was employed to ensure that PCG had adequate contact with MB, a contaminant in the high-salinity wastewater, promoting the rapid absorption and transport of the contaminant by the interfacial solar evaporator to the gas–liquid phase. Due to its accessibility and widespread presence, MB has frequently been used as a target pollutant for evaluating photocatalytic efficiency. After a 5 h adsorption–desorption equilibrium under dark conditions, the degradation efficiency of all samples was measured. This study utilized deionized water as the water source and conducted a series of experiments under simulated sunlight.

## 3. Results and Discussion

### 3.1. Fabrication of PCG Evaporators

**Figure 1:**
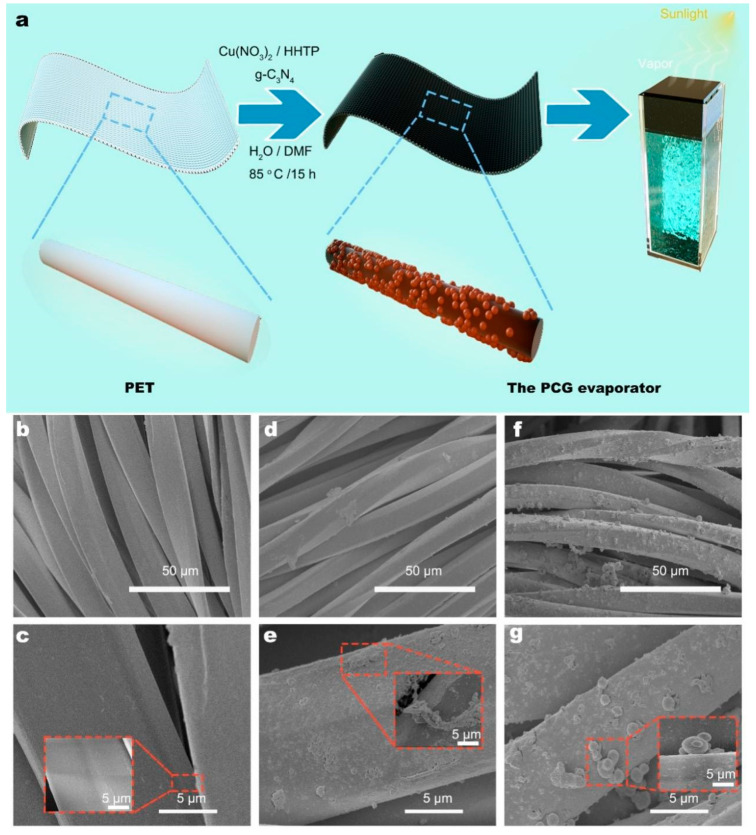
(**a**) Schematic fabrication of the PCG evaporators. SEM images of the (**b**,**c**) PET fabric, (**d**,**e**) PET@Cu-MOF evaporator and (**f**,**g**) PCG evaporator.

The schematic fabrication process of the PCG evaporator is illustrated in Figure 1a. First, the nonwoven PET fabric was cleaned with anhydrous ethanol and distilled water until no impurities remained on the surface. Scanning electron microscopy (SEM) analysis revealed that the surfaces of the PET microfibers were smooth and free of contaminants (Figure 1b). Subsequently, Cu (NO_3_)_2_, HHTP and 0.1 g of g-C_3_N_4_ were dispersed in a DMF/H_2_O solution and stirred uniformly. During the solvothermal process, the reaction between HHTP and Cu^2+^ resulted in the deposition of Cu-MOF crystals on the PET microfibers. Cu-MOFs exhibited strong adsorption of g-C_3_N_4_, facilitating its loading onto the PET microfibers (Figure 1f). The morphological characterization of Cu-MOFs and g-C_3_N_4_ was analyzed by SEM (Figure 1b–g). As shown in Figure 1c, the PET fabric consists of numerous microfibers with smooth, impurity-free surfaces and an inter-fiber gap of approximately 5 μm, providing excellent water transport properties. As depicted in Figure 1d, the surface of the PET@Cu-MOF evaporator is covered with abundant Cu-MOF crystals. The Cu-MOF crystals present a rod-like structure with diameters ranging from 100 to 800 nm. Numerous irregular rod-like micro/nanostructures are present on the surface, increasing the surface roughness of PET@Cu-MOFs and forming abundant porous structures. These features help enhance sunlight refraction, creating microscopic “photothermal traps” (Figure 1e). As shown in Figure 1f, the PCG surface not only carries numerous rod-shaped Cu-MOF crystals but also contains abundant regular spherical micro/nanostructures. Figure 1g shows regular spherical structures with diameters ranging from 0.8 to 1.5 μm. This aggregation of g-C_3_N_4_ is attributed to self-deposition. According to a previous literature report, (ClCN)_3_ and DCD were used as precursors to form g-C_3_N_4_ via a solvothermal reaction [18,20,22].

The elemental distribution on the surface of the PCG evaporator was analyzed using SEM-EDS. Carbon (C), nitrogen (N) and copper (Cu) are present on the surface of the PCG evaporator, as depicted in Figure S2. The surface of the PCG evaporator was loaded with a large amount of evenly distributed g-C_3_N_4_. Further evidence from SEM-EDS mapping and SEM confirmed the presence of g-C_3_N_4_ on its surface and successfully validated that g-C_3_N_4_ was loaded onto the surface of the PET through the adsorption force of Cu-MOFs. g-C_3_N_4_ has a nanoscale structure and density similar to that of the aqueous solution (2.0 g/cm^3^), which makes it difficult to separate and recycle it. This method provides a new approach to address the challenge of recycling and reusing g-C_3_N_4_.

**Figure 2:**
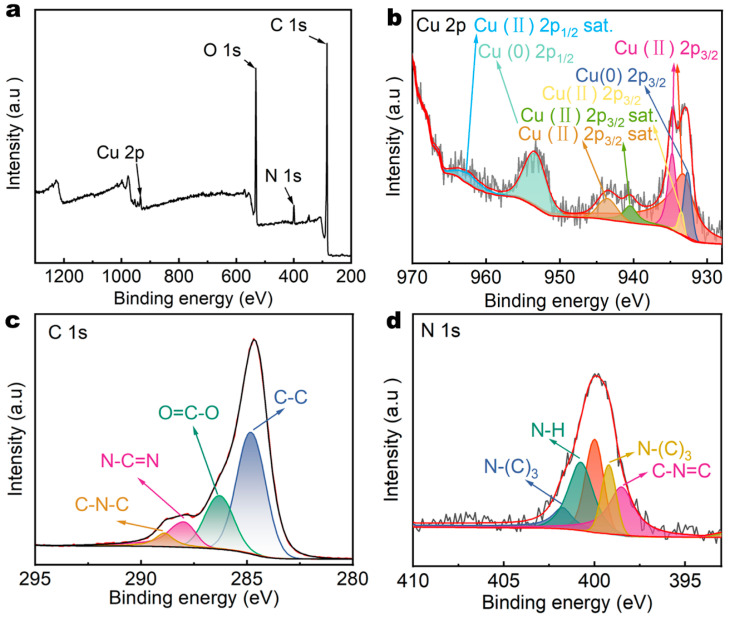
(**a**) XPS survey spectrum of the PCG evaporator. (**b**) Cu 2p, (**c**) C 1s and (**d**) N 1s XPS spectra of the PCG evaporator.

To further demonstrate the successful loading of g-C_3_N_4_ and Cu-MOFs onto PET, the surface chemical composition of the PCG evaporator was analyzed using X-ray photoelectron spectroscopy (XPS). The XPS survey spectrum indicated the presence of Cu 2p (933.38 eV), N 1s (399.98 eV), C 1s (284.88 eV) and O 1s peaks (Figure 2a), originating from g-C_3_N_4_ and Cu-MOFs. The chemical state of Cu was analyzed using the spectrum of Cu 2p with a high resolution (Figure 2b). Cu 2p primarily exists in two forms, Cu (0) and Cu (II). The peaks at 932.68 eV and 953.48 eV correspond to Cu (0) 2p_3/2_ and Cu (0) 2p_1/2_, which is likely due to the reduction of Cu (II) during synthesis of Cu-MOFs [24]. Due to coordination between Cu (II) and HHTP, the Cu (II) 2p_3/2_ peak splits into two peaks at 934.78 eV and 933.58 eV, indicating the formation of Cu-MOFs [25]. The satellite peak at 962.38 eV is attributed to Cu (II) 2p_1/2_ [26]. The satellite peaks at 943.48 eV and 940.48 eV are attributed to Cu (II) 2p_3/2_. These satellite peaks result from charge transfer excitation from O 2p to Cu 2p, indicating the formation of Cu-O bonds [27]. The high-resolution C 1s spectrum was used to analyze the chemical state of C (Figure 2c). The dominant peaks are observed at 284.88 eV, 286.38 eV, 287.98 eV and 288.98 eV. The peak at 284.88 eV is associated with the presence of adventitious C–C coordination [28]. The peak at 286.38 eV corresponds to the ester bond O=C-O arising from the polyester structure [28]. The peak at 287.98 eV is assigned to C with sp^2^ hybridization in the form of N-C=N [28]. The peak at 288.98 eV is assigned to the sp^2^ carbon atoms bonded to nitrogen [15]. The high-resolution N 1s spectrum was used to analyze the chemical state of N (Figure 2d). The dominant peaks appeared at 398.5 eV, 400.78 eV, 401.88 eV and 399.18 eV. The peak at 398.5 eV is assigned to the sp^2^ C=N bond in the triazine ring, the peak at 399.18 eV is associated to the graphitic nitrogen, the peak at 400.78 eV is associated to the N–H and the peak at 401.88 eV indicates the existence of the bridging sp^3^ N atoms in N–(C)_3_ groups [24].

### 3.2. Solar Absorption and Photothermal Effect of the PCG Evaporators

**Figure 3:**
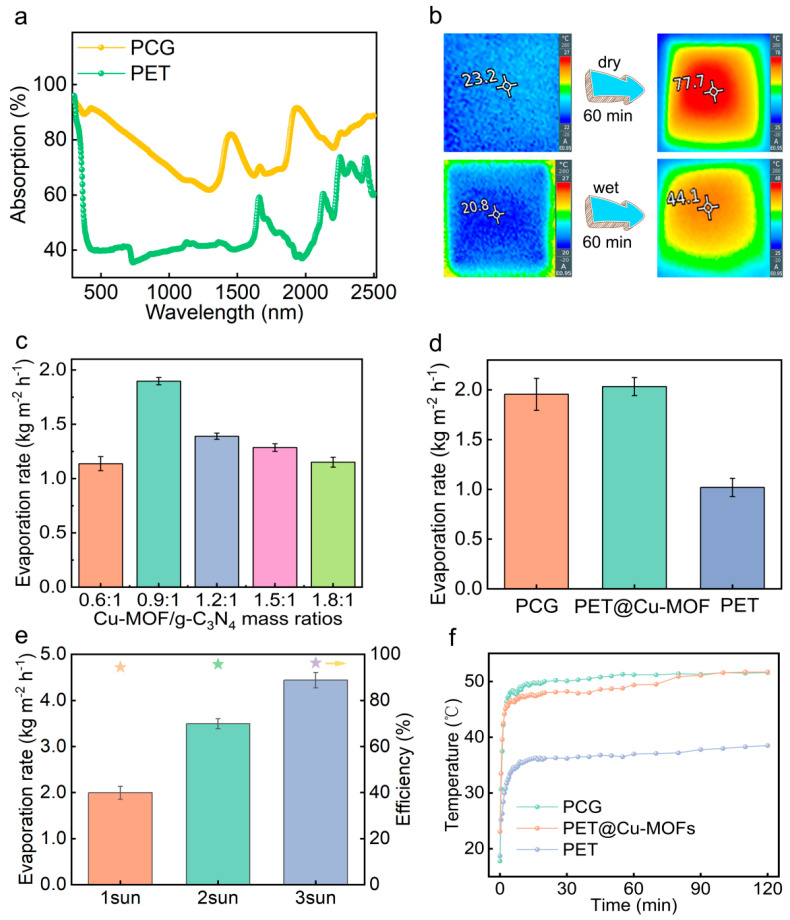
(**a**) UV-Vis-NIR absorption spectra of the PET fabric and PCG evaporator. (**b**) The surface temperature of the dry and wet PCG evaporator after illumination under 1 sun for 60 min. (**c**) Effect of the Cu-MOF/g-C_3_N_4_ mass ratio during preparation of the PCG evaporators on their evaporation rates under 1 sun in 3.5 wt% NaCl solution. (**d**) Evaporation rates of PET, PET@Cu-MOFs and PCG under 1 sun. (**e**) Evaporation efficiency and rate of the PCG evaporator under different sunlight intensity conditions. (**f**) Changes in the surface temperature of various evaporators during solar evaporation under 1 sun. The stars in (**e**) indicates the evaporation efficiency of the PCG evaporator under different salt solutions. The data in (**c**–**e**) are presented as mean ± SD, *n* = 3.

The rough surface microstructures of solar evaporators can induce a light-trapping effect, enhancing the multiple scattering of incident light [29,30,31,32]. This results in increased light absorbance and reduced reflectivity of the evaporators. Figure 3a shows the light absorption of the PCG evaporator is 84.4%, significantly higher than that of PET (39.0%) across the broad solar spectrum of 250–2500 nm. The result illustrates that the PCG evaporator has excellent light absorbing properties. In the dry state, the PCG evaporator can rapidly heat up to 77.7 °C from 23.2 °C within 60 min under 1 sun (Figure 3b), demonstrating its excellent light-responsive capacity. In the wet state, the PCG evaporator reaches 44.1 °C from 20.8 °C within 60 min under 1 sun (Figure 3b). This indicates that the surface temperature of the evaporator is more likely to reach thermal equilibrium in the wet state, where the process is more stable. The superior water transfer performance of the PCG evaporator plays a crucial role in maintaining temperature balance.

Water mass changes were observed using a real-time laboratory monitoring system. The evaporation performance of the PCG evaporator was evaluated by measuring the change in water mass (Figure S3a). The PCG evaporator was wrapped around the top surface and four side walls of a cubic polystyrene foam with low thermal conductivity (~0.02 W m^−1^ K^−1^). The time-dependent mass changes of 3.5 wt% NaCl solution under 1 sun are depicted in Figure S3b. The rate of evaporation was precisely calculated from the slope of the mass–time variation curve.

The proportionality between photothermal material (Cu-MOFs) and photocatalytic material (g-C_3_N_4_) influences the photothermal performance and solar absorption. The PCG evaporator with a Cu-MOF/g-C_3_N_4_ mass ratio of 0.9:1 exhibits superior photothermal performance compared to other compositions (Figure 3c). The equilibrium temperature of its surface is slightly higher than that of other configurations (Figure S4). Under 1 sun, the surface temperature rapidly rises to 49.2 °C within 20 min and stabilizes at 52.2 °C after 60 min during solar evaporation of a 3.5 wt% NaCl solution. Consequently, the evaporation rate of 3.5 wt% NaCl solution reaches a peak of 1.89 kg m^−2^ h^−1^, outperforming the PCG evaporators with other chemical compositions.

The evaporation rates of the PCG and PET@Cu-MOF evaporators for 3.5 wt% NaCl solution are significantly higher than that of PET (Figure 3d), due to the photothermal properties of Cu-MOFs and the rough microstructure of the evaporators. The PCG evaporator achieves a maximum evaporation rate of 1.955 kg m^−2^ h^−1^ at 1 sun. This rate is comparable to that of PET@Cu-MOFs (2.03 kg m^−2^ h^−1^), indicating that the introduction of g-C_3_N_4_ has a minimal impact on the evaporation rate. Additionally, its evaporation efficiency is 94.4% (Figure 3e). Under 1 sun, the surface equilibrium temperature of the PCG evaporator (49.5 °C) is much higher than that of PET (37 °C) during solar evaporation of 3.5 wt% NaCl solution (Figure 3f). The incorporation of Cu-MOFs markedly enhances solar absorption and efficient photothermal conversion. Under 3 suns, PCG evaporators evaporate in a 3.5 wt% NaCl solution at 4.44 kg m^−2^ h^−1^, exhibiting high photothermal efficiency and light absorption (Figure 3e).

### 3.3. Salt Resistance of PCG Evaporators

**Figure 4:**
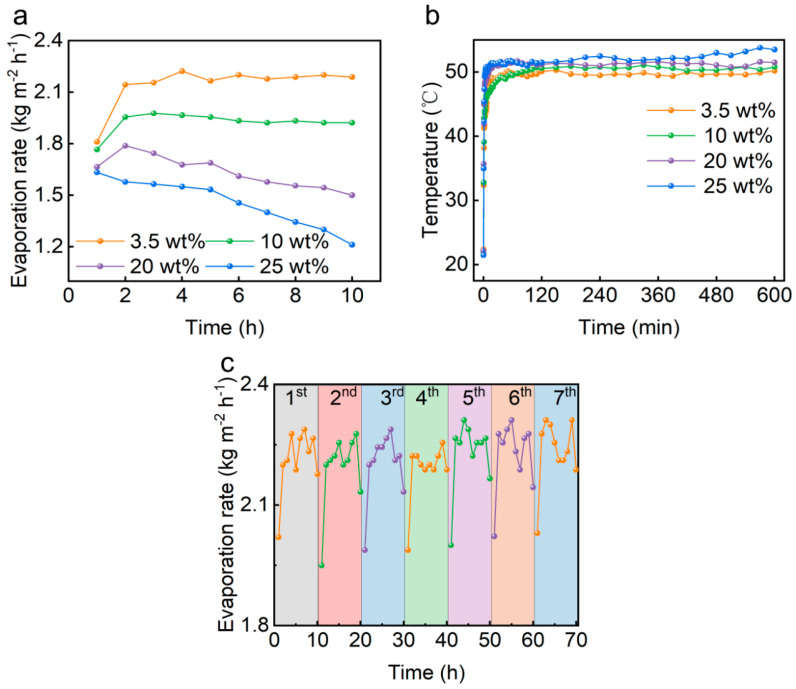
(**a**) Changes in evaporation rates of various brines during continuous 10 h solar evaporation using the PCG evaporator under 1 sun. (**b**) Changes in the surface temperature of the PCG evaporator during continuous 10 h solar evaporation of various NaCl solutions under 1 sun. (**c**) Changes in evaporation rate of the 3.5 wt% NaCl solution during continuous 7 days of solar evaporation using the PCG evaporator under 1 sun with 10 h irradiation per day.

**Figure 5:**
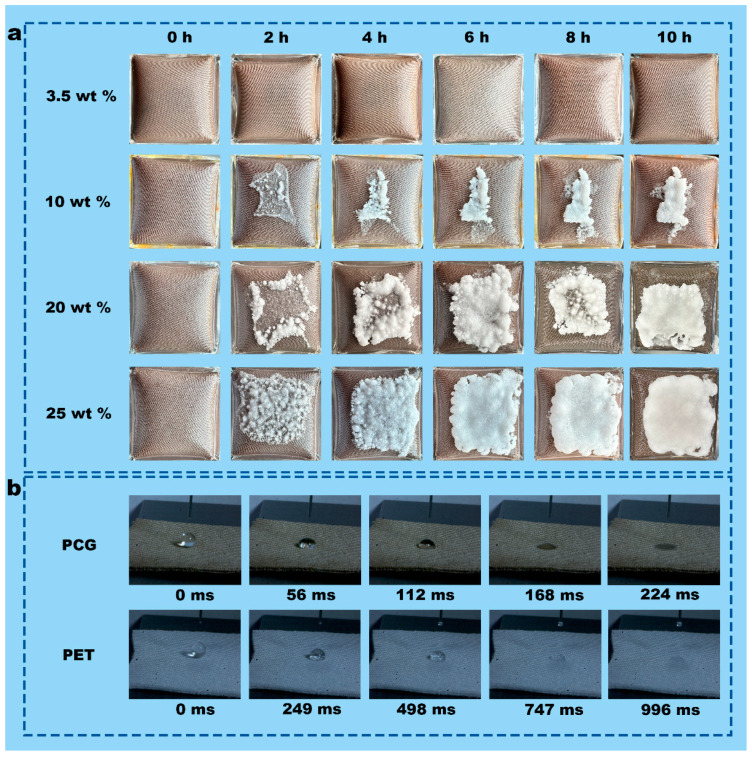
(**a**) Salt deposition on the top surface of the PCG evaporator during continuous 10 h solar evaporation of various NaCl solutions under 1 sun. (**b**) PET fabrics and PCG evaporators: dynamic wetting characteristics.

Solar evaporation tests were conducted in simulated brine solutions with different concentrations of NaCl to evaluate the salt resistance of the PCG evaporator. As shown in Figure 4a, in 3.5 wt% NaCl solution under 1 sun, the evaporation rate was 1.81 kg m^−2^ h^−1^ at 1 h. And the average evaporation rate was 2.14 kg m^−2^ h^−1^ for 10 h. The increase in the average evaporation rate is attributed to the preheating of water in the chamber as the evaporation time extends. Under 1 sun, the evaporation rate of the PCG evaporator decreased from an initial 1.63 kg m^−2^ h^−1^ to 1.21 kg m^−2^ h^−1^ at 10 h in 25 wt% NaCl solution (Figure 4a). The evaporation rate showed a decreasing trend with the increase in NaCl concentration because many salt crystals accumulated on the top surface of the solar-driven interface evaporator, which over time formed a “salt crust” that prevented the photothermal layer from receiving sunlight with the increase in NaCl concentration (Figure 5a). The formation of the “salt crust” during the evaporation process in the 2D evaporator followed an interesting pattern. As shown in Figure 5a, during evaporation of the 20 wt% NaCl solution, the compact salt crystals were preferentially aggregated on the edge and gradually distributed throughout the photothermal layer, forming a “salt crust” as the evaporation time progressed, which is consistent with previous studies [30].

A large area of accumulated salt crystals on the surface of the evaporator not only reduced the evaporation performance but also increased the surface temperature. As shown in Figure 4b, the surface temperature of the PCG evaporator (53.8 °C) in 20 wt% NaCl solution was higher than the highest surface temperature of the PCG evaporator (50.2 °C) in 3.5 wt% NaCl solution. This is because the surface temperature of the PCG evaporator in 20 wt% NaCl solution was elevated by the transfer of energy from a multitude of salt crystals. However, the evaporation rate of the PCG evaporator in 20 wt% NaCl solution was lower than the evaporation rate in 3.5 wt% NaCl solution (Figure 4a).

Despite serious salt deposition during solar evaporation of the 25 wt% NaCl solution, the salt crystals on the top surface of the PCG evaporator almost completely disappear after standing for 24 h under dark conditions (Figure S5) because the PCG evaporator has excellent water transfer performance. The liquid-climbing experiment and the dynamic wetting analysis demonstrate the excellent water transport performance (Figure 5b and Movie S1). In the liquid-climbing test, the water transfer rate of the PCG evaporator is 4.5 m s^−1^, substantially higher than that of PET (0.5 m s^−1^). Moreover, high-speed camera analysis of the dynamic wetting characteristics reveals that the PCG evaporator absorbs water droplets much more rapidly than PET (Figure 5b). At 224 ms, the PCG evaporator fully absorbs the water droplet, whereas PET takes 996 ms to do so. This is because of the presence of numerous MOF particles with excellent absorbability on the PET microfibers of the PCG evaporator.

A long-term cyclic evaporation performance test was conducted in 3.5 wt% NaCl solution for 7 days with continuous illumination of 10 h in each day (Figure 4c). The PCG evaporator maintained an average evaporation rate of approximately 2.14 kg m^−2^ h^−1^. Notable fluctuations in the evaporation rate were observed, which can be attributed to intermittent increases in air flow, leading to significant energy loss and a temporary reduction in evaporation rate. These results demonstrate the excellent evaporation stability of the PCG evaporator.

### 3.4. Photocatalytic Degradation of MB with Simulated Sunlight

**Figure 6:**
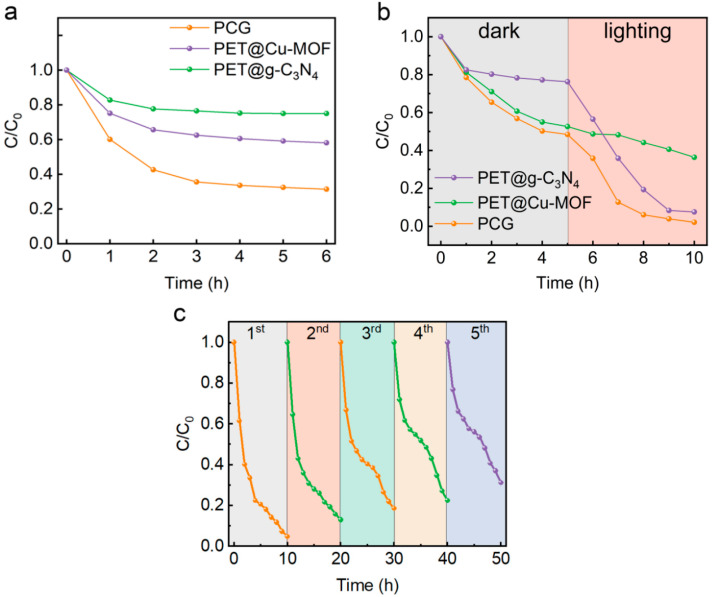
(**a**) Changes in MB concentration during adsorption using PET@g-C_3_N_4_, PET@Cu-MOFs and PCG in simulated high-salinity wastewater with MB (3.5 wt% NaCl, 10 mg/L MB) in a dark environment. (**b**) Changes in MB concentration in simulated high-salinity wastewater with MB (3.5 wt% NaCl, 10 mg/L MB) under dark (5 h) and then illumination (5 h, 1 sun) conditions in the presence of PET@g-C_3_N_4_, PET@Cu-MOFs and PCG. (**c**) Cyclic removal of MB in simulated high-salinity wastewater with MB (3.5 wt% NaCl, 10 mg/L MB) using the PCG evaporator under 1 sun.

The solar interface evaporator PCG, coupled with photocatalytic function, exhibited excellent evaporation performance during the evaporation process. To investigate the interrelationship between the photothermal and photocatalytic properties of photothermal materials (PET@Cu-MOFs), photocatalytic materials (PET@g-C_3_N_4_) and photothermal-photocatalytic hybrid materials (PCG) in simulated high-salinity wastewater and to evaluate the application potential of the PCG evaporator in treating high-salinity wastewater, we prepared simulated high-salinity wastewater with MB as the contaminant (3.5 wt% NaCl, 10 mg/L MB). As shown in Figure 6a, PET@Cu-MOFs, PET@g-C_3_N_4_ and PCG reached adsorption equilibrium for MB after 5 h in a dark environment. Due to the strong adsorption capability of Cu-MOFs, PET@Cu-MOFs and PCG demonstrated significantly higher adsorption capacities for MB than PET@g-C_3_N_4_.

Figure 6b shows the removal performance of PET@Cu-MOFs, PET@g-C_3_N_4_ and PCG for MB in simulated high-salinity wastewater under dark (5 h) and then illumination (5h, 1 sun) conditions. Among them, the PCG evaporator demonstrated the best removal performance, with a removal ratio of 98%, significantly higher than those of PET@Cu-MOFs (63.6%) and PET@g-C_3_N_4_ (92.5%). Note that under illumination conditions, the removal rate of PCG (46.4%) is lower than that of PET@g-C_3_N_4_ (68.7%) but much higher than that of PET@Cu-MOFs (16.1%). The comparison of removal performance under illumination and dark conditions indicates that Cu-MOFs primarily exhibit a photothermal effect under illumination conditions, whereas the PCG evaporator shows a synergistic effect involving photothermal performance, photocatalytic degradation and adsorption. This confirms the excellent potential of the PCG evaporator for treating high-salinity wastewater.

The removal efficiency of the PCG evaporator for MB remained high with the increase in the degradation cycles, although the removal ratio gradually decreased from an initial 95.4% to a final 68.9% (Figure 6c). This phenomenon is attributed to the continuous deposition of MB on the surface of the PCG evaporator during each degradation cycle (Figure S6), reducing its adsorption capacity. The results preliminarily demonstrate the excellent potential of the PCG solar evaporator, coupled with photocatalytic functionality, for treating high-salinity wastewater.

### 3.5. Freshwater Collection by Simultaneous Solar Evaporation and Photocatalytic Degradation in Outdoor Conditions

**Figure 7:**
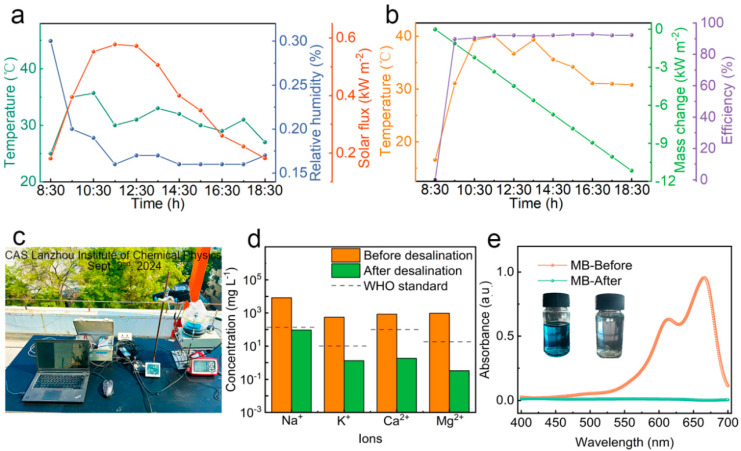
(**a**) Temperature, solar flux and relative humidity of outdoor air at CAS Lanzhou Institute of Chemical Physics on 2 September 2024. (**b**) Mass change, surface temperature and evaporation rate of PCG evaporator during solar-driven interfacial evaporation of simulated high-salinity wastewater with MB (3.5 wt% NaCl, 10 mg/L MB) under natural sunlight (closed system). (**c**) Photo of outdoor closed system device for solar evaporation. (**d**) Concentrations of four primary ions in simulated high-salinity wastewater with MB and collected freshwater. (**e**) UV-Vis spectra of simulated high-salinity wastewater with MB (3.5 wt% NaCl, 10 mg/L MB) before and collected freshwater after solar purification. The insets show the solutions before and after treatment.

An outdoor interfacial solar evaporation experiment was conducted under natural sunlight to evaluate the performance of the solar evaporator PCG, coupled with photocatalytic function, in treating high-salinity wastewater in natural conditions. During the experiment, ambient temperature and relative humidity (RH) varied gradually with changes in solar radiation intensity (Figure 7a). The outdoor interfacial solar evaporation experiment was conducted from 8:30 to 18:30 on 2 September 2024, and the evaporation rate of the PCG evaporator and the temperature changes on the top surface were recorded, with solar radiation intensity ranging from 0.18 kW m^−2^ to 0.57 kW m^−2^. The experiment was conducted at the CAS Lanzhou Institute of Chemical Physics. At 11:30, the surface temperature of the PCG evaporator reached 40 °C under 0.57 suns (Figure 7b). During the continuous 10 h interfacial solar evaporation, the average evaporation rate of the PCG evaporator under natural sunlight was 1.24 kg m^−2^ h^−1^, lower than the evaporation rate under simulated 1 sun (1.98 kg m^−2^ h^−1^) conditions. This discrepancy is mainly attributed to the lower solar radiation intensity under natural sunlight.

The PCG evaporator was further investigated by building an all-in-one closed device for collecting freshwater (Figure 7c). The appearance of vapor mist was observed after a period of 10 min, while condensed water was evident on the conical glass plate after 60 min. The effect of gravity resulted in the condensed water accumulating within the container (Figure S7). Within 10 h of consecutive interfacial solar evaporation of the high-salinity wastewater with MB under natural sunlight illumination, freshwater was collected at a rate of 1.24 kg m^−2^ h^−1^ for a total of 12.3 kg m^−2^, which meets the minimum daily water intake for adults. Moreover, the evaporation efficiency of the PCG evaporator is as high as 91.7% (Figure 7b).

The concentrations of four typical ions in the collected freshwater were measured by inductively coupled plasma optical emission spectrometry (ICP-OES). Ion concentrations of Na^+^, Ca^2+^, K^+^ and Mg^2+^ in the high-salinity MB solution were significantly reduced from 8250, 800.4, 550.6 and 955.3 mg L^−1^ to 93.1, 1.816, 1.304 and 0.324 mg L^−1^, respectively (Figure 7d). The ion concentrations in the collected water were far below the limits defined in the drinking water standards defined by WHO, as indicated by the dashed lines in Figure 7d. Moreover, the organic pollutant MB in the collected freshwater is not detectable, as measured using a UV-Vis spectrophotometer (Figure 7e), indicating the excellent photocatalytic performance of the PCG evaporator under natural sunlight. These results demonstrate excellent potential of the PCG solar evaporator, coupled with photocatalytic functionality, for treating high-salinity organic wastewater to obtain clean water resources.

## 4. Conclusions

In summary, we successfully developed a highly efficient solar evaporator that synergistically integrates photothermal evaporation, photocatalytic degradation and adsorption for high-salinity organic wastewater treatment. Under 1 sun, the evaporator achieved an impressive evaporation rate of 1.95 kg m^−2^ h^−1^ and an evaporation efficiency of 94.43%. These outstanding results can be attributed to its superhydrophilic surface for rapid water transport. After treatment with the solar evaporator, the ion concentrations in the brine were significantly below the limits defined in the WHO drinking water standards. Moreover, the evaporator exhibited excellent adsorption and photocatalytic degradation performance for MB molecules. Notably, under real sunlight conditions, the organic pollutant MB in the collected freshwater is not detectable, and a high freshwater production rate of 1.24 kg m^−2^ h^−1^ is observed. This work provides a promising solution for obtaining clean water from high-salinity wastewater and natural seawater, with broad potential applications across diverse environments.

## Data Availability

The data presented in this study are available on request from the corresponding author.

## Supplementary Materials

The following supporting information can be downloaded at: https://www.mdpi.com/article/10.3390/nano15080632/s1, Figure S1: g-C_3_N_4_ sample image; Figure S2: Elemental mapping of the PET@g-C_3_N_4_/Cu-MOFs (PCG); Figure S3: (a) the laboratory real-time monitoring system, (b) Time-dependent mass changes of 3.5 wt% saline solution during solar evaporation under 1sun in the presence of PET, PET@Cu-MOFs and PCG evaporators; Figure S4: Changes in the surface temperature of various PCG evaporators with different Cu-MOF/g-C_3_N_4_ mass ratios during solar evaporation of 3.5 wt% saline solution under 1 sun; Figure S5: Photograph of the PCG evaporator after continuous 10 h solar evaporation of 25 wt% saline solution under 1 sun and then being left in darkness for 24 h; Figure S6: Photographs of the PCG evaporator after (a) the 2nd degradation cycle and (b) the 5th degradation cycle; Figure S7: Photographs of the all-in-one closed device for collecting freshwater during outdoor solar evaporation for (a) 10 min and (b) 60 min; Figure S8: Photographs of laboratory self-assembled MB degradation test device. Movie S1: Video of the liquid-climbing experiment of PET and PCG (FASTCAM Mini UX100, Photron, Japan).

## References

[1] Zhou W., Naila A., Chen H., Muhammad S.I., Naveed M., Shahid H., Matiullah S., Syed Z.H.N., Muhammad R., Naeem S. (2022). Interfacial Photothermal Heat Accumulation for Simultaneous Salt Rejection and Freshwater Generation; an Efficient Solar Energy Harvester. Nanomaterials.

[2] Miao H., Muneerah A., Areej S.A., Naila A., Muhammad A., Muhammad Y., Muhammad S.I.Y.-Z.L., Qiang L. (2023). Strontium-Cobaltite-Based Perovskite (SrCoO_3_) for Solar-Driven Interfacial Evaporation Systems for Clean Water Generation. Nanomaterials.

[3] Xu X., Zhao Q., Liu Q., Qiu J., Yuan S., Wu Z., Yang R., Cao J., Wang L., Xu J. (2023). A Bilayered Wood-Poly(3,4-ethylenedioxythiophene):Polystyrene Sulfonate Hydrogel Interfacial Evaporator for Sustainable Solar-Driven Sewage Purification and Desalination. Nanomaterials.

[4] Akhtar N., Syakir Ishak M.I., Bhawani S.A., Umar K. (2021). Various Natural and Anthropogenic Factors Responsible for Water Quality Degradation: A Review. Water.

[5] Dong X., Gao S., Li S., Zhu T., Huang J., Chen Z., Lai Y. (2021). Bioinspired structural and functional designs towards interfacial solar steam generation for clean water production. Mater. Chem. Front..

[6] Cao S., Jiang Q., Wu X., Ghim D., Gholami D.H., Chou P.-I., Jun Y.-S., Singamaneni S. (2019). Advances in solar evaporator materials for freshwater generation. J. Mater. Chem. A.

[7] Pistocchi A., Bleninger T., Breyer C., Caldera U., Dorati C., Ganora D., Millan M.M., Paton C., Poullis D., Herrero F.S. (2020). Can seawater desalination be a win-win fix to our water cycle?. Water Res..

[8] Hu R., Zhang J., Kuang Y., Wang K., Cai X., Fang Z., Huang W.H., Chen G., Wang Z. (2019). A Janus evaporator with low tortuosity for long-term solar desalination. J. Mater. Chem. A.

[9] Ni G., Zandavi S.H., Javid S.M., Boriskina S.V., Cooper T.A., Chen G. (2018). A salt-rejecting floating solar still for low-cost desalination. Energy Environ. Sci..

[10] Zhao L., Yang Q., Guo W., Liu H., Ma T., Qu F. (2019). Co_2.67_S_4_-Based Photothermal Membrane with High Mechanical Properties for Efficient Solar Water Evaporation and Photothermal Antibacterial Applications. ACS Appl. Interfaces.

[11] Qu B., Li P., Bai L., Qu Y., Li Z., Zhang Z., Zheng B., Sun J., Jing L. (2023). Atomically Dispersed Zn-N_5_ Sites Immobilized on g-C_3_ N_4_ Nanosheets for Ultrasensitive Selective Detection of Phenanthrene by Dual Ratiometric Fluorescence. Adv. Mater..

[12] Li Z., Xu X., Sheng X., Lin P., Tang J., Pan L., Kaneti Y.V., Yang T., Yamauchi Y. (2021). Solar-Powered Sustainable Water Production: State-of-the-Art Technologies for Sunlight-Energy-Water Nexus. ACS Nano.

[13] Cui Y., Ding Z., Fu X., Wang X. (2021). Construction of conjugated carbon nitride nanoarchitectures in solution at low temperatures for photoredox catalysis. Angew. Chem. Int. Ed. Engl..

[14] Mu X., Chen L., Qu N., Yu J., Jiang X., Xiao C., Luo X., Hasi Q. (2023). MXene/polypyrrole coated melamine-foam for efficient interfacial evaporation and photodegradation. J. Colloid Interface Sci..

[15] Rtimi S., Pulgarin C., Kiwi J. (2017). Recent Developments in Accelerated Antibacterial Inactivation on 2D Cu-Titania Surfaces under Indoor Visible Light. Coatings.

[16] Liu H., Li X., Ma Z., Sun M., Li M., Zhang Z., Zhang L., Tang Z., Yao Y., Huang B. (2021). Atomically Dispersed Cu Catalyst for Efficient Chemoselective Hydrogenation Reaction. Nano Lett..

[17] Han Y., Xu Z., Gao C. (2013). Ultrathin Graphene Nanofiltration Membrane for Water Purification. Adv. Funct. Mater..

[18] Liu F., Fan M., Liu X., Chen J. (2024). One-Pot Synthesis of Cellulose-Based Carbon Aerogel Loaded with TiO_2_ and g-C_3_N_4_ and Its Photocatalytic Degradation of Rhodamine B. Nanomaterials.

[19] Li J., Yu X., Zhu Y., Fu X., Zhang Y. (2021). 3D-2D-3D BiOI/porous g-C_3_N_4_/graphene hydrogel composite photocatalyst with synergy of adsorption-photocatalysis in static and flow systems. J. Alloys Compd..

[20] Lu Y., Liu C., Zheng L., Chen F., Qian J., Meng X., Chen Z., Zhong S., He B. (2025). N_3_C-Defect-Tuned g-C_3_N_4_ Photocatalysts: Structural Optimization and Enhanced Tetracycline Degradation Performance. Nanomaterials.

[21] Lv B., Li S., Yu Y., Liu Y., Xu Y., Fan X. (2024). A 2.5-Dimensional biomimetic dual-functional bifacial-evaporator for high efficient evaporation and water purification. J. Hazard. Mater..

[22] Xiong T., Cen W., Zhang Y., Dong F. (2016). Bridging the g-C_3_N_4_ Interlayers for Enhanced Photocatalysis. ACS Catal..

[23] Zimmerman J.L.R.W., Khabashesku V.N., Margrave J.L. (2001). Synthesis of Spherical Carbon Nitride Nanostructures. Nano Lett..

[24] Chen K., Li L., Li B., Yang Y., Zhu K., Zhang J. (2024). Simultaneous Fresh Water Collection and Li^+^ Selective Adsorption Enabled by A Salt-Resistant Separated Solar Evaporator. Adv. Func. Mater..

[25] Gao Z., Guan J., Wang M., Liu S., Chen K., Liu Q., Chen X. (2024). A novel laccase-like Cu-MOF for colorimetric differentiation and detection of phenolic compounds. Talanta.

[26] Wang F.-F., Sun W.-Y. (2024). Cu-MOF and CuBi Double-Perovskite Composites for Selective CO_2_ Electroreduction to HCOOH. ACS Sustain. Chem. Eng..

[27] Majdoub A., Majdoub M., Zaitan H. (2024). g-C_3_N_4_/CuO loaded polyester fabric as effective Fenton-like dip-catalyst for the oxidation of dyes. J. Water Process. Eng..

[28] Zeng L., Deng D., Zhu L., Wang H., Zhang Z., Yao Y. (2023). Biomass photothermal structures with carbonized durian for efficient solar-driven water evaporation. Energy.

[29] Zuo Z., Zhu F., Wang L., Wang Z., Zhao J., Ji Z., An M., Ye Y.N., Yu W., Wang Z. (2024). Trapping waste metal ions in a hydrogel/coal powder composite for boosting sewage purification via solar-driven interfacial water evaporation with long-term durability. Chem. Eng. J..

[30] Huang X.P., Li L.X., Chen K., Zhang J.P. (2024). Scalable Superhydrophilic Solar Evaporators for Long-Term Stable Desalination, Fresh Water Collection and Salt Collection by Vertical Salt Deposition. ChemSusChem.

[31] Huang X., Li L., Zhao X., Zhang J. (2023). Highly Salt-Resistant interfacial solar evaporators based on Melamine@Silicone nanoparticles for stable Long-Term desalination and water harvesting. J. Colloid Interface Sci..

[32] Wagner M., Andrew Lin K.Y., Oh W.D., Lisak G. (2021). Metal-organic frameworks for pesticidal persistent organic pollutants detection and adsorption—A mini review. J. Hazard. Mater..

