# Supplementary material for: Flexible Solar Interfacial Evaporators with Photocatalytic Function for Purification of High-Salinity Organic Wastewater
Source: Nanomaterials (Basel). 2025 Apr 21;15(8):632. doi: 10.3390/nano15080632 (PMC12029355; doi:10.3390/nano15080632)
Supplement: Supplementary file 1 [file nanomaterials-15-00632-s001.zip › nanomaterials-3587461-supplementary.pdf]

Supporting Information

Flexible Solar Interfacial Evaporators with Photocatalytic Function for Purification of High-Salinity Organic Wastewater

Yucheng Li [1,2], Xia Zhao [1,*], Tao Hu [2], Lingxiao Li [3], Xiaopeng Huang [4] and Junping Zhang [2,*]

[1] Department of Environmental Engineering, College of Petrochemical Engineering, Lanzhou University of Technology, Lanzhou 730050, China; lao17629086530@163.com
[2] Research Center of Resource Chemistry and Energy Materials, Lanzhou Institute of Chemical Physics,
Chinese Academy of Sciences, Lanzhou 730000, China; hutao@licp.cas.cn
[3] State Key Laboratory of Featured Metal Materials and Life-Cycle Safety for Composite Structures, School of Resources, Environment and Materials, Guangxi University, Nanning 530004, China; lilx@gxu.edu.cn
[4] Department of Chemical Engineering, College of Petrochemical Engineering, Lanzhou University of
Technology, Lanzhou 730050, China; 18419210628@139.com
* Correspondence: zhaoxia@lut.edu.cn (X.Z.); jpzhang@licp.cas.cn (J.Z.)

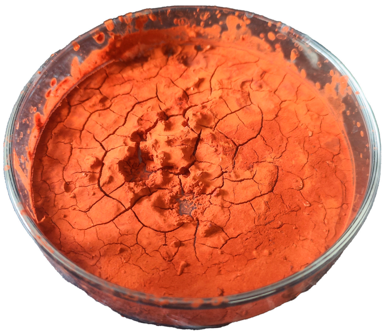

Figure S1. Photograph of g-C_3N_4.

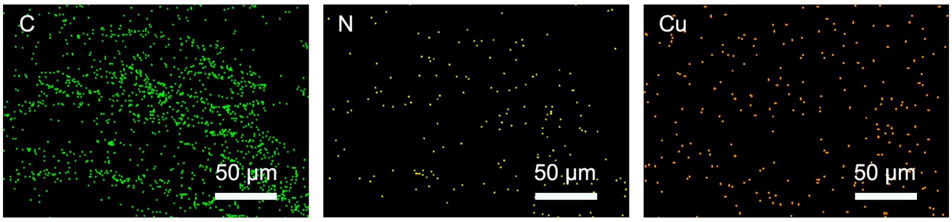

Figure S2. Elemental mapping of the PCG evaporator.

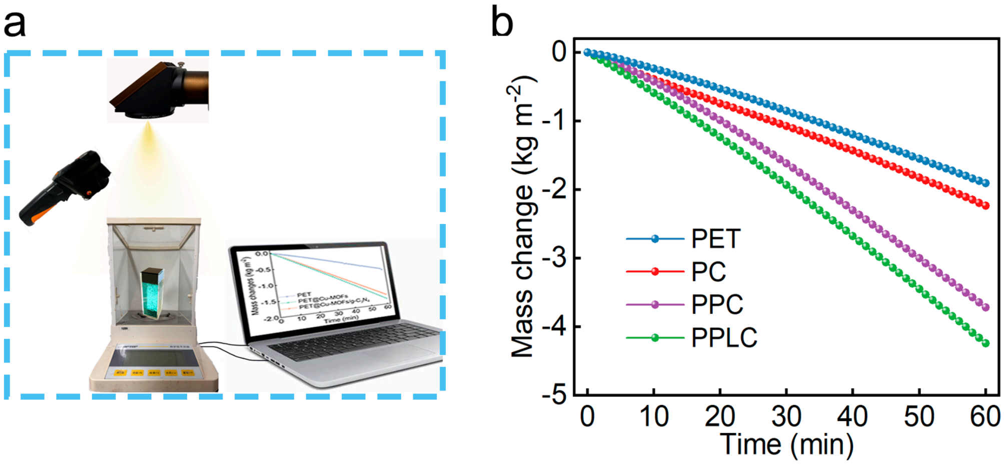

Figure S3. (a) The laboratory real-time monitoring system. (b) Time-dependent mass changes of 3.5 wt% saline solution during solar evaporation under illumination of 1sun in the presence of PET, PET@Cu-MOFs and PCG evaporators.

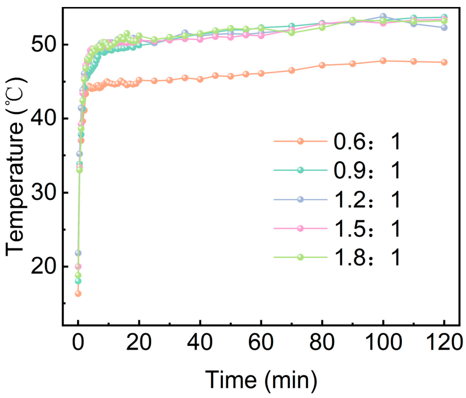

Figure S4. Changes of top surface temperature of various PCG evaporators with different Cu-MOF/g-C_3N_4 mass ratios during solar evaporation of 3.5 wt% NaCl solution under 1 sun.

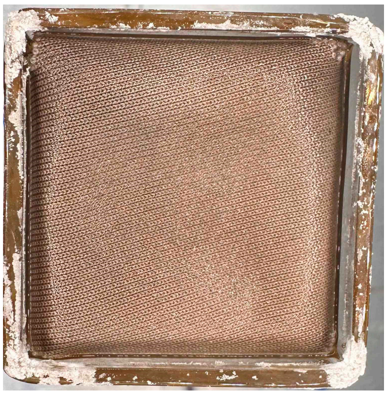

Figure S5. Photograph of the PCG evaporator after continuous 10 h solar evaporation of 25 wt% NaCl solution under 1 sun and then being left in darkness for 24 h.

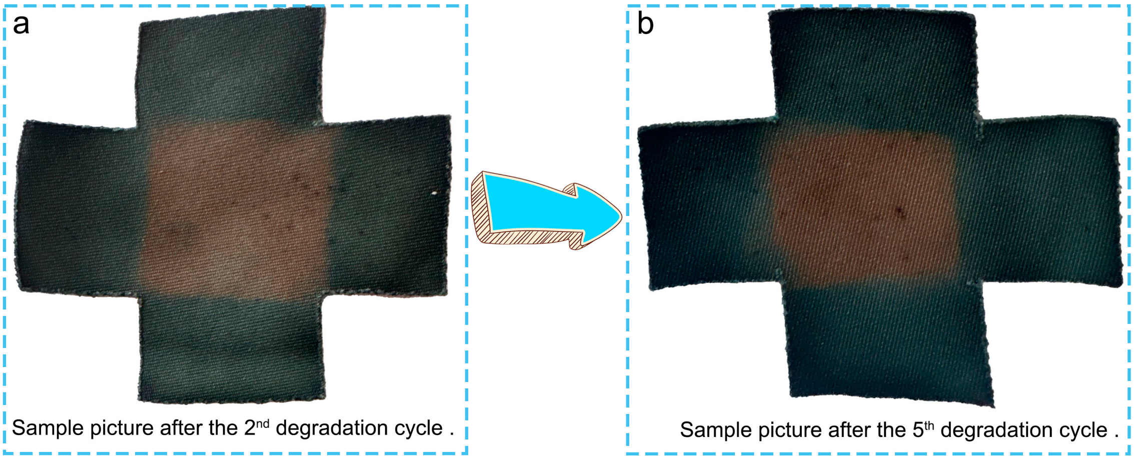

Figure S6. Photographs of the PCG evaporator after (a) the 2nd degradation cycle and (b) the 5th degradation cycle.

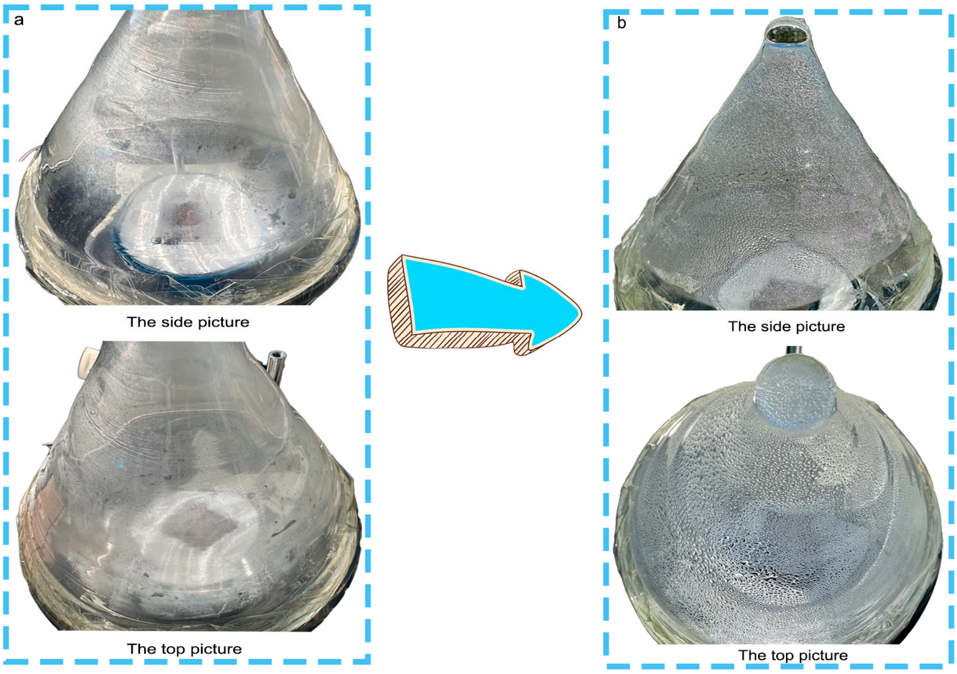

Figure S7. Photographs of the all-in-one closed device for collecting fresh water during outdoor solar evaporation for (a) 10 min and (b) 60 min.

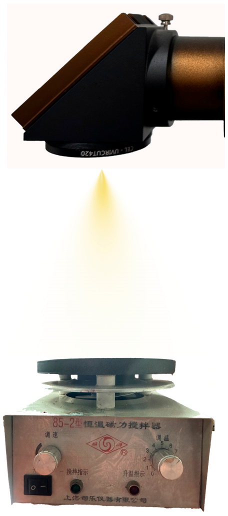

Figure S8. Photographs of laboratory self-assembled MB degradation test device.

Movie S1. Video of the liquid-climbing experiment of PET and PCG (FASTCAM Mini UX100, Photron, Japan).
